# Prediction models for early diagnosis of actinomycotic osteomyelitis of the jaw using machine learning techniques: a preliminary study

**DOI:** 10.1186/s12903-022-02201-6

**Published:** 2022-05-06

**Authors:** Sun-Gyu Choi, Eun-Young Lee, Ok-Jun Lee, Somi Kim, Ji-Yeon Kang, Jae Seok Lim

**Affiliations:** 1 Department of Oral and Maxillofacial Surgery, Hankook General Hospital, Danjae-ro 106, Sangdang-gu, Cheongju, South Korea; 2 Department of Oral and Maxillofacial Surgery, College of Medicine and Medical Research Institute, Chungbuk National University, Chungdae-ro 1, Seowon-Gu, Cheongju, Chungbuk 28644 South Korea; 3 Department of Oral and Maxillofacial Surgery, Chungbuk National University Hospital, 776, 1Sunhwan-ro, Seowon-gu, Cheongju, Chungbuk 28644 South Korea; 4 Department of Pathology, Chungbuk National University Hospital, 776, 1Sunhwan-ro, Seowon-gu, Cheongju, Chungbuk 28644 South Korea; 5 Dental Clinic Center, Chungnam National University Hospital, Sejong, South Korea; 6 Department of Oral and Maxillofacial Surgery, College of Medicine, Chungnam National University, Daejeon, South Korea

**Keywords:** Actinomycosis, Machine learning, Osteomyelitis

## Abstract

**Background:**

This study aimed to develop and validate five machine learning models designed to predict actinomycotic osteomyelitis of the jaw. Furthermore, this study determined the relative importance of the predictive variables for actinomycotic osteomyelitis of the jaw, which are crucial for clinical decision-making.

**Methods:**

A total of 222 patients with osteomyelitis of the jaw were analyzed, and *Actinomyces* were identified in 70 cases (31.5%). Logistic regression, random forest, support vector machine, artificial neural network, and extreme gradient boosting machine learning methods were used to train the models. The models were subsequently validated using testing datasets. These models were compared with each other and also with single predictors, such as age, using area under the receiver operating characteristic (ROC) curve (AUC).

**Results:**

The AUC of the machine learning models ranged from 0.81 to 0.88. The performance of the machine learning models, such as random forest, support vector machine and extreme gradient boosting was significantly superior to that of single predictors. Presumed causes, antiresorptive agents, age, malignancy, hypertension, and rheumatoid arthritis were the six features that were identified as relevant predictors.

**Conclusions:**

This prediction model would improve the overall patient care by enhancing prognosis counseling and informing treatment decisions for high-risk groups of actinomycotic osteomyelitis of the jaw.

**Supplementary Information:**

The online version contains supplementary material available at 10.1186/s12903-022-02201-6.

## Background

Actinomycotic osteomyelitis of the jaw (AOJ) is a rare, sporadic chronic infection characterized by a granulomatous and suppurative lesion located primarily in the jaw bone. Typically, AOJ presents as a slowly progressing painless intraosseous lesion, evolving into soft tissue abscesses with draining sinus tracts on the skin surface or oral mucosa, which at times exudes a typical thick yellow exudate with characteristic sulfur granules [1, 2]. The incidence of *Actinomyces* infection in mandible is 53.6%, followed by chin (13.3%), maxilla (5.7%), and temporomandibular joint (TMJ) (0.3%) [3]. Individuals with poor oral hygiene, history of mucosal trauma, male gender, diabetes, immunosuppression, and malnutrition have an increased risk for developing actinomycosis [1]. *Actinomyces israelii* is the most prevalent species isolated in AOJ cases and requires resection of the sequestrated bone and a prolonged course of antibiotics [2, 4–6].

Early diagnosis plays an important role in preventing the serious consequences of progressive osteomyelitis, such as pathologic fracture and deformity [1, 7]. However, since AOJ is an infectious disease, it is difficult to diagnose based on clinical and radiological features. Microscopic examination and bacterial culture of the abscess are the gold standard method to diagnose AOJ [1, 4, 8]. However, administration of oral antibiotics before surgery leads to frequent false-negative results of the cultures in patients with osteomyelitis [6, 9, 10]. In addition, the surgical specimen used for the pathologic examination cannot be obtained until the necrotic bone is removed, thereby delaying the diagnosis. Thus, a new predictive approach using machine learning (ML) that can reflect the simultaneous analysis of various reported predisposing factors, including poor oral hygiene (such as dental caries, odontogenic infection), mucosal trauma (such as dental extraction), antiresorptive agent, gender, and diabetes mellitus, is required [8, 11].

In recent years, an increasing amount of research applying ML techniques to medical classification has been conducted [12]. Their recent extensive application can be attributed to the increased availability of electronic health records [13]. However, there are very few published studies applying ML to osteomyelitis caused by an infection as direct identification or isolation of the infecting organism from a specimen of osteomyelitis may be laborious and time-consuming. Therefore, the purpose of this study was to develop and validate five ML models designed to predict AOJ to help provide guidelines for clinical decision-making and more effective treatment.

## Methods

All experiments were performed in accordance with the guidelines and regulations approved by the Institutional Review Board (IRB No. 2020-06-002-0003) of Chungbuk National University Hospital and informed consent was obtained from all participants.

### Study population and data collection

**Fig. 1:**
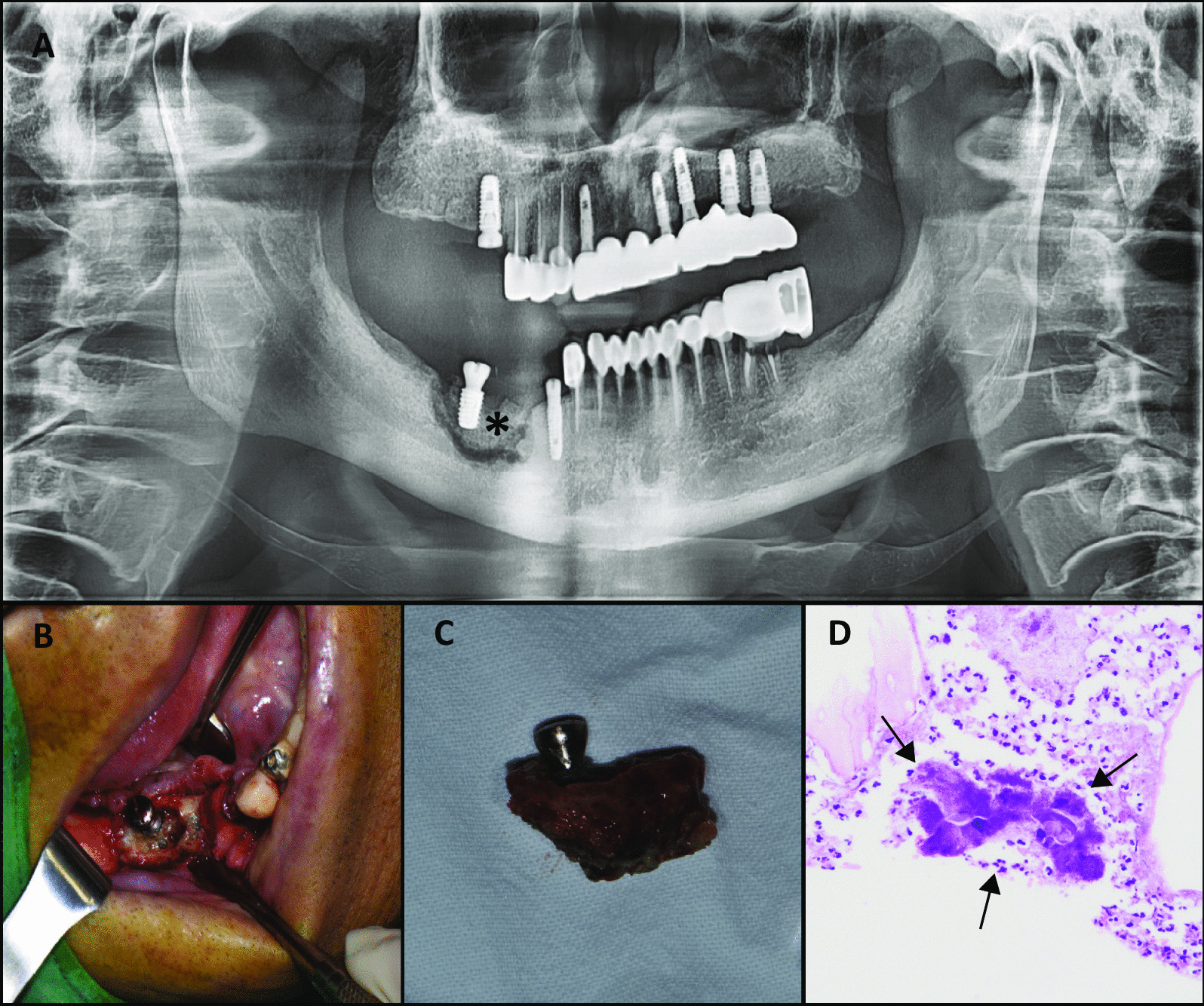
Representative case of actinomycotic osteomyelitis of the jaw (AOJ). **a** Preoperative panoramic view showing radiolucent and radiopaque areas in the right mandibular premolar region below the implant (asterisk). **b** Intraoperative clinical view showing sequestrum in the right mandibular premolar region. **c** Excised sequestrum and neighboring implant. **d** Histological examination showed the basophilic sulfur granule (black arrow) with radiating filament surrounded by mixed inflammatory cell infiltration (Hematoxylin–Eosin, × 400), consistent with AOJ. *AOJ* actinomycotic osteomyelitis of the jaw

We retrospectively enrolled patients with osteomyelitis of the jaw treated in the Department of Oral and Maxillofacial Surgery, Chungbuk National University Hospital, South Korea, between January 2015 and June 2020. A representative case is shown in Fig. 1. Only patients who underwent sequestrectomy were included (Fig. 1a, b). The exclusion criteria were as follows: (1) multiple osteomyelitis of the jaw, (2) history of radiation therapy to the jaw, (3) patient loss during follow-up, and (4) incomplete medical records. The medical records of the patients were reviewed retrospectively to collect data, including age, gender, presumed causes, anatomical site, comorbidities, use of antiresorptive agents (ARA), use of antithrombotic agents, and recurrence. In total, 578 patient records were reviewed, and 222 patients were finally selected.

### Histological analysis

The removed sequestrums were embedded in paraffin, cut into slices of 2 μm thickness, and stained using hematoxylin and eosin. A trained pathologist examined the slides for pathognomonic features of actinomycosis, such as sulfur granules. Photographs were taken of slides visualized by light microscopy. (Fig. 1c, d).

### Machine learning

A schematic of the study design is shown in Additional file 1: Fig. S1 (see Additional file 1). Five ML methods, namely logistic regression (LR), random forest (RF), artificial neural network, support vector machine (SVM), and extreme gradient boosting (XGB) using the caret package provided in the R statistical software version 3.6.3 and R studio, (R Foundation for Statistical Computing, Vienna, Austria) were used to generate the prediction model [14–16]. The study design consisted of random splitting of the input dataset into training (n = 156; 70% of 222 patients) and testing (n = 66, 30% of 222 patients) datasets while maintaining equal proportions of the class ratios in each split. We developed five final ML models to predict actinomycotic infection in the training dataset by tuning the hyper-parameters using the caret package provided with the R statistical software (see Additional file 1: Table S1, Additional file 1, Additional file 2). We used five-fold cross-validation with 10 repeats to prevent overfitting. The Boruta algorithm based on random forest model was used to calculate the relative feature importance, which was provided in arbitrary units [17].

### Statistical analysis

Statistical analysis was conducted using the R statistical software version 3.6.3 and R studio [14, 15]. The frequency tables were analyzed using Student’s t-test and the χ^2^ test, as appropriate. The association between the variables and the AOJ-positive group was calculated using univariate regression analysis. The correlation between the two variables was demonstrated using Spearman's correlation analysis. *P* values < 0.05 (two-sided) were considered statistically significant. Five models were compared with each other and also with single predictors, such as age, using area under the receiver operating characteristic (ROC) curve (AUC) plotted using ggplot2 that is open-source data visualization package implemented in R [18]. ROC curves of single predictors in testing dataset including the age, gender, presumed causes, anatomical site, comorbidities, use of antiresorptive agents (ARA), use of antithrombotic agents, and recurrence were plotted. The AUCs were compared using the Delong test. The optimal threshold was calculated as the point closest to the top-left part of the plot. The performance metrics, including the accuracy, sensitivity, specificity, positive predictive value (PPV) and negative predictive value (NPV) were obtained.

## Results

The baseline characteristics of the patients are shown in Table 1. The age, proportion of females, the proportion of dental extraction and implants in the AOJ-positive group were significantly higher than that in the AOJ-negative group. Moreover, patients diagnosed with hypertension (HTN), cancer, patients using ARA, and recurrence were more common in the AOJ-positive group than in the AOJ-negative group. Interestingly, there was no recurrence in AOJ-negative group. In the correlation analysis, the AOJ-positive group highly correlated with three variables, namely patients using ARA (ρ = 0.53, *p* < 0.001), age (ρ = 0.37, *p* < 0.001), and presumed causes (ρ = − 0.41, *p* < 0.001) (Additional file 1: Fig. S2, see Additional file 1).

**Table 1:** Baseline characteristics of all patients included in our analysis

Dependent: Actinomycosis	Negative	Positive	*P*
Gender			
Female	62 (40.8)	44 (62.9)	0.004
Male	90 (59.2)	26 (37.1)	
Age (years)			
Mean (SD)	62.3 (16.1)	75.0 (12.3)	< 0.001
Presumed causes			
Odontogenic infection	136 (89.5)	26 (37.1)	< 0.001
Dental extraction	5 (3.3)	27 (38.6)	
Implant	3 (2.0)	7 (10.0)	
Unknown	8 (5.3)	10 (14.3)	
Anatomical site			
Maxilla posterior	40 (26.3)	18 (25.7)	0.266
Maxilla anterior	9 (5.9)	4 (5.7)	
Mandible posterior	99 (65.1)	42 (60.0)	
Mandible anterior	4 (2.6)	6 (8.6)	
Comorbidities			
Hypertension	68 (44.7)	45 (64.3)	0.010
Diabetes mellitus	40 (26.3)	19 (27.1)	1.000
Heart disease	35 (23.0)	14 (20.0)	0.741
Renal disease	18 (11.8)	7 (10.0)	0.861
Liver disease	9 (5.9)	1 (1.4)	0.250
Cerebral disease	13 (8.6)	1 (1.4)	0.083
Malignancy	7 (4.6)	10 (14.3)	0.025
Rheumatoid arthritis	1 (0.7)	4 (5.7)	0.061
Antiresorptive agents	16 (10.5)	41 (58.6)	< 0.001
Antithrombotic agents	44 (28.9)	25 (35.7)	0.392
Recurrence		9 (12.9)	< 0.001

*SD* standard deviation

**Fig. 2:**
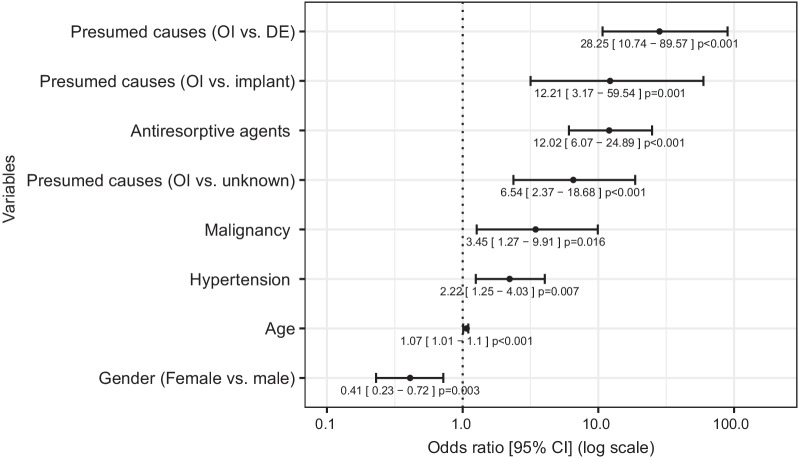
Univariate regression analysis to identify variables associated with the AOJ-positive group. Forest plots indicate the odds ratios and confidence intervals of the variables associated with the AOJ-positive group. Black dots indicate the odds ratios for the variables (*p* < 0.05) and error bars indicate 95% confidence intervals. *AOJ* actinomycotic osteomyelitis of the jaw, *CI* confidence interval, *DE* dental extraction, *OI* odontogenic infection

We performed a univariate regression analysis to identify the single independent feature associated with the AOJ-positive group (Fig. 2, Table 2). Presumed causes (odontogenic infection vs. dental extraction) (odds ratio [OR] 28.25; 95% confidence interval [CI] 10.74–89.57, *p* < 0.001), ARA (OR 12.02; 95% CI 6.07–24.89, *p* < 0.001), malignancy (OR 3.45; 95% CI 1.27–9.91, *p* = 0.016), HTN (OR 2.22; 95% CI 1.25–4.03, *p* = 0.007), age (OR 1.07; 95% CI 1.04–1.10, *p* < 0.001) and gender (female vs. male) (OR 0.41; 95% CI 0.23–0.72, *p* = 0.003) were significantly associated with the AOJ-positive group.

**Table 2:** Univariate regression analysis

Label	Levels	Negative	Positive	OR (univariable)
Gender	Female	62 (58.5)	44 (41.5)	–
	Male	90 (77.6)	26 (22.4)	0.41 (0.23–0.72, *p* = 0.003)
Age (years)	Mean (SD)	62.3 (16.1)	75.0 (12.3)	1.07 (1.04–1.10, *p* < 0.001)
Presumed causes	Odontogenic infection	136 (84.0)	26 (16.0)	–
	Dental extraction	5 (15.6)	27 (84.4)	28.25 (10.74–89.57, *p* < 0.001)
	Implant	3 (30.0)	7 (70.0)	12.21 (3.17–59.54, *p* = 0.001)
	Unknown	8 (44.4)	10 (55.6)	6.54 (2.37–18.68, *p* < 0.001)
Anatomical site	Maxilla posterior	40 (69.0)	18 (31.0)	–
	Maxilla anterior	9 (69.2)	4 (30.8)	0.99 (0.24–3.48, *p* = 0.985)
	Mandible posterior	99 (70.2)	42 (29.8)	0.94 (0.49–1.86, *p* = 0.862)
	Mandible anterior	4 (40.0)	6 (60.0)	3.33 (0.85–14.45, *p* = 0.088)
Comorbidities	Hypertension	68 (60.2)	45 (39.8)	2.22 (1.25–4.03, *p* = 0.007)
	Diabetes mellitus	40 (67.8)	19 (32.2)	1.04 (0.54–1.96, *p* = 0.897)
	Heart disease	35 (71.4)	14 (28.6)	0.84 (0.41–1.65, *p* = 0.614)
	Renal disease	18 (72.0)	7 (28.0)	0.83 (0.31–2.01, *p* = 0.687)
	Liver disease	9 (90.0)	1 (10.0)	0.23 (0.01–1.26, *p* = 0.168)
	Cerebral disease	13 (92.9)	1 (7.1)	0.15 (0.01–0.80, *p* = 0.075)
	Malignancy	7 (41.2)	10 (58.8)	3.45 (1.27–9.91, *p* = 0.016)
	Rheumatoid arthritis	1 (20.0)	4 (80.0)	9.15 (1.32–180.87, *p* = 0.050)
Antiresorptive agents		16 (28.1)	41 (71.9)	12.02 (6.07–24.89, *p* < 0.001)
Antithrombotic agents		44 (63.8)	25 (36.2)	1.36 (0.74–2.48, *p* = 0.312)

*OR* odds ratio, *SD* standard deviation

**Fig. 3:**
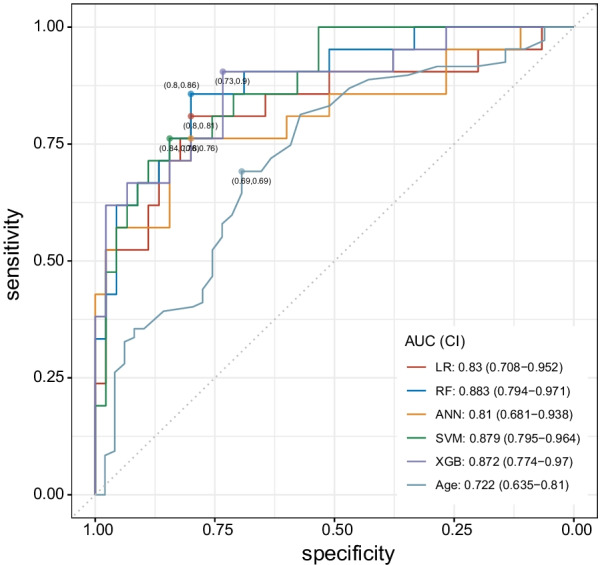
ROC curves of machine learning (ML) models and single predictor. AUC of RF, SVM, and XGB are significantly higher than single predictor (age). *ANN* artificial neural network, *AUC* area under the ROC curve, *CI* confidence interval, *LR* logistic regression, *ML* machine learning, *RF* random forest, *ROC* receiver operating characteristic, *SVM* support vector machine, *XGB* extreme gradient boosting

Subsequently, we developed a prediction model using ML techniques. A schematic diagram of the prediction model development is shown in Additional file 1: Fig. S1 (see Additional file 1). The ratio of AOJ-positive patients was 31.5% (70/222), which was consistent with the imbalanced data (Table 1). Therefore, we applied the oversampling methods to rebalance the training dataset. We subsequently tested all models using the testing dataset. The AUCs of all models were above 0.8, indicating that all models performed effectively in the testing dataset. The performance of ML, such as RF, SVM, and XGB, was significantly superior to that of the single predictor (such as age) (Fig. 3, Additional file 1: Fig. S3, Additional file 1: Fig. S4, Additional file 1: Fig. S5, Additional file 1: Table S2; see Additional file 1).

**Fig. 4:**
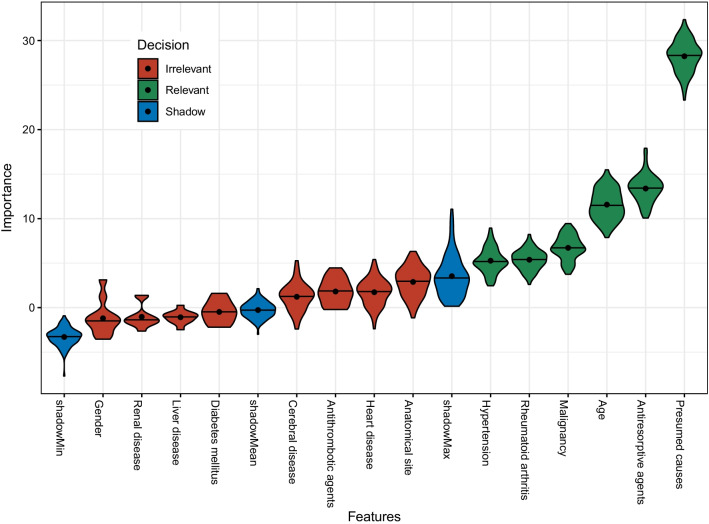
Relative feature importance computed using the Boruta algorithm. Blue violin plots correspond to the minimal, average, and maximum Z scores of a shadow attribute. Red and green violin plots represent the Z scores of the rejected and confirmed attributes, respectively. Black dots and horizontal lines inside each violin plot represent the mean and median values, respectively. All features that received a lower relative feature importance than that of the shadow feature were defined as irrelevant for prediction

Lastly, the relative importance of all features was calculated using the Boruta algorithm [17]. Presumed causes, ARA, age, malignancy, rheumatoid arthritis, and HTN were the six features determined to be relevant in predicting AOJ-positive patients (Fig. 4). The performance of the prediction models, including accuracy, sensitivity, and specificity, PPV, and NPV is shown in Table 3.

**Table 3:** Accuracy, sensitivity and specificity of the prediction models

Model	Accuracy	Sensitivity	Specificity	PPV	NPV
LR	0.80	0.81	0.80	0.65	0.90
RF	0.82	0.86	0.80	0.67	0.92
ANN	0.79	0.76	0.80	0.64	0.88
SVM	0.82	0.76	0.84	0.70	0.88
XGB	0.79	0.90	0.73	0.61	0.94
Age	0.69	0.69	0.69	0.83	0.51

*LR* logistic regression, *RF* random forest, *ANN* artificial neural network, *SVM* support vector machine, *XGB* extreme gradient boosting, *PPV* positive predictive value, *NPV* negative predictive value

## Discussion

Herein, we developed ML-based models designed to predict the presence of *Actinomyces* in the jaw bone, which has not been previously attempted, to the best of our knowledge. We also included the performance metrics with the ROC curve and feature importance to enhance the interpretability of the ML models. All five prediction models exhibited comparable accuracy, and the value of the AUC (0.81 to 0.88) indicated excellent categorization regarding the predictive performance [19].

Multiple factors seem to affect the development of AOJ simultaneously. Therefore, clinicians often find it difficult to integrate these factors and their complex relationship with AOJ to guide treatment decisions-making. In our study, all ML models performed better than single predictors, namely age, suggesting that these models helped us analyze combinations of features to predict AOJ. It is noteworthy that combining only a few variables significantly increased the performance of the ML models, suggesting that a large number of variables is not essential to generate a good predictive model.

In recent years, ML approaches have gained popularity as a tool for all healthcare analysis, especially for medical image classification [20]. The greater availability of electronic medical records, as well as advances in hardware and software, have contributed to their recent widespread use. [21–23]. Despite these improvements in these approaches to classification tasks, current ML models, especially deep neural network, still operate like black boxes and fail to provide interpretations for their predictions [24]. It is also true that there are simple interpretable models such as LR. In the LR model, the coefficients helped us understand the cause of individual predictions. In our study, we used the Boruta algorithm based on the RF model to calculate the feature importance, which would allow clinicians to understand the relative importance of the variables involved in the overall prediction. Notably, presumed causes (such as extraction) were revealed as the most important risk factor in the relative feature importance calculated by the Boruta algorithm and regression analysis simultaneously. Since *Actinomyces* is a normal inhabitant of the oral cavity and lacks tissue-decomposing enzymes (such as hyaluronidases), mechanical trauma is the prerequisite that allows these endogenous microbial pathogens entry through the mucosal barrier and into the jaw leading to actinomycosis [4]. In line with this, our study revealed that the proportion of dental extractions and implants were significantly higher in the AOJ-positive group than that in the AOJ-negative group.

In addition, ARA was revealed as the second risk factor following presumed causes in the relative feature importance calculated by the Boruta algorithm. Previous studies have reported that *Actinomyces* species could be detected in about 80% of the samples from patients with medication-related osteonecrosis of the jaw (MRONJ) using histological techniques [10, 11, 25]. Consistent with this, our analysis showed that *Actinomyces* was present in 41 of 57 (71.9%) patients taking ARA. However, non-MRONJ patients showed a relatively low detection rate of *Actinomyces* (17.5%, 29 of 165) from the bone specimens, implying that *Actinomyces* was associated with the pathogenesis of MRONJ. Accurate causal inference and the role of *Actinomyces* underlying the development of AOJ remains elusive due to the lack of experimental validation in our study. It is still possible that actinomycosis is an opportunistic infection to pre-existent local osteomyelitis of the jaw bone. In the future, prospective studies investigating the microbiome originating from osteomyelitis of the jaw bone are needed to better understand the role of *Actinomyces* in the development of AOJ.

Treatment standards for invasive actinomycosis have been developed and adapted from various studies and are based on prolonged antimicrobial treatment (such as amoxicillin with clavulanic acid) for 2–6 months combined with surgery [4, 5, 11, 26]. Notably, recurrence was seen in only nine AOJ-positive patients in our study. Among those, six were administered antibiotics for less than 2 months, indicating the importance of extending antibiotic therapy. All patients with recurrence were completely cured after the prolonged administration of antibiotics and removal of the foci of infection, including sequestrectomy and excision of the granulation tissue until the sound bone was exposed. The capability for causal inference between recurrence and AOJ was limited due to the retrospective nature of this study.

A diagnosis of actinomycosis is best achieved by culture. However, the sensitivity of the culture is reduced significantly by the administration of antibiotics before sample collection [6, 9, 10]. In addition, special handling is needed to culture anaerobic organisms [27, 28]. Therefore, histological examination is also preferred. *Actinomyces* species can be detected reliably in the affected bone specimens because of their morphologic appearance with staining [10]. In our analysis, *Actinomyces* species were histologically detected in 31.5% of the bone specimens, but there may still be an underestimation of the factual frequency of osteomyelitis associated with this infection. Recent developments in molecular methods such as 16 s rRNA target sequencing have revolutionized new approaches for the rapid detection of microorganisms, including those difficult to culture [29]. The sensitivity of the histological evaluation of clinical specimens for microbes is generally lower than microbiome analyses using sequencing technology since the latter involves an amplification step that increases the number of diagnostic targets. Thus, in the future, microbial detection using sequencing technology will likely be used for more accurate diagnosis.

This study has several limitations. The retrospective and cross-sectional nature of this study restricted causal inference. Further prospective studies should investigate the applicability of ML models for future prediction by transforming these retrospective data into a longitudinal research design. In addition, the overall performance of the ML techniques was comparable to that of LR. This result is mainly caused by the use of a dataset composed of categorical variables in this study. While the results are significant, we entitled this study as preliminary because of the limited number of patients and features. Furthermore, our analysis facilitated only speculation regarding the pathogenesis of AOJ with respect to various features owing to the lack of experimental validation in the ML technique. Recent advances in sequencing technologies and culture-independent methods have further elucidated the associations between the oral microbiome and oral health and disease state [29–31]. Additional studies using sequencing technologies are needed in the future to understand the microbial composition of the lesion in AOJ patients and achieve a rapid diagnosis.

## Conclusions

Five prediction models exhibited comparable accuracy, and the range of the AUC results of 0.81 to 0.88 indicate good categorization in terms of predictive performance. The performance of the ML models, such as RF, SVM, and XGB was significantly superior to that of single predictors. Six features, such as presumed causes, antiresorptive agents, age, malignancy, hypertension, and rheumatoid arthritis were identified as relevant predictors. Hence, our prediction model, which considered various factors together as one complex, would improve the overall patient care by enhancing the prognosis counseling and informing treatment decisions to high-risk groups of AOJ.

## Supplementary Information

**Additional file 1: Fig. S1.** Schematic of study design. **Fig. S2.** Correlation plot of all features. **Fig. S3.** Receiver operating characteristic curves plotted from testing dataset using single predictors. **Fig. S4.** ROC curves of each ML model. **Fig. S5.** Calibration plot of the prediction models. **Table S1.** Optimal parameter of selected model. **Table S2.** Calculated probability of the difference between the area under the receiver operating characteristic curve.

**Additional file 2.** Model selection during training.

## Data Availability

The datasets used and/or analyzed during the study are available from the corresponding author on reasonable request.

## Abbreviations

AOJ: Actinomycotic osteomyelitis of the jaw
ARA: Antiresorptive agents
AUC: Area under the ROC curve
CI: Confidence interval
HTN: Hypertension
LR: Logistic regression
ML: Machine learning
MRONJ: Medication-related osteonecrosis of the jaw
OR: Odds ratio
RF: Random forest
ROC: Receiver operating characteristic
SVM: Support vector machine
TMJ: Temporomandibular joint
XGB: Extreme gradient boosting
