# Supplementary material for: Prediction models for early diagnosis of actinomycotic osteomyelitis of the jaw using machine learning techniques: a preliminary study
Source: BMC Oral Health. 2022 May 6;22:164. doi: 10.1186/s12903-022-02201-6 (PMC9074201; doi:10.1186/s12903-022-02201-6)
Supplement: Supplementary file 1 — Additional file 1: Fig. S1. Schematic of study design. Fig. S2. Correlation plot of all features. Fig. S3. Receiver operating characteristic curves plotted from testing dataset using single predictors. Fig. S4. ROC curves of each ML model. Fig. S5. Calibration plot of the prediction models. Table S1. Optimal parameter of selected model. Table S2. Calculated probability of the difference between the area under the receiver operating characteristic curve. [file 12903_2022_2201_MOESM1_ESM.pdf]

ADDITIONAL FILES 1

1. Supplementary Appendix Figure 1. Schematic of study design
2. Supplementary Appendix Figure 2. Correlation plot of all features
3. Supplementary Appendix Figure 3. Receiver operating characteristic curves plotted from testing dataset using single predictors
4. Supplementary Appendix Figure 4. ROC curves of each ML model
5. Supplementary Appendix Figure 5. Calibration plot of the prediction models
6. Supplementary Appendix Table 1. Optimal parameter of selected model
7. Supplementary Appendix Table 2. Calculated probability of the difference between the area under the receiver operating characteristic curve

Supplementary Appendix Figure 1. Schematic of studty design.

LR: logistic regression, RF: random forest, ANN: artificial neural network, SVM: support vector machine, XGB: extreme gradient boosting

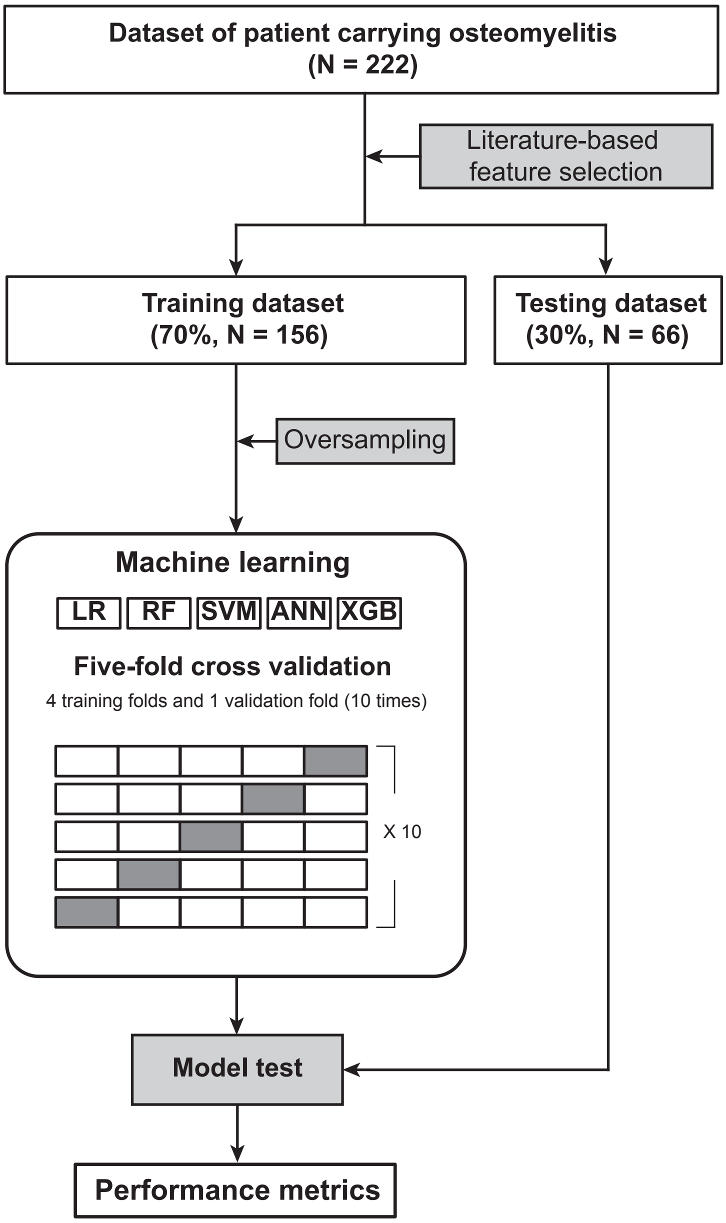

Supplementary Appendix Figure 2. Correlation plot of all feature.

The colors represent the degree of pairwise correlation regarding the Spearman's rank correlation coefficient (rho). The darker blue color indicate a stronger positive correlation, whereas the darker red represents a stronger negative correlation.

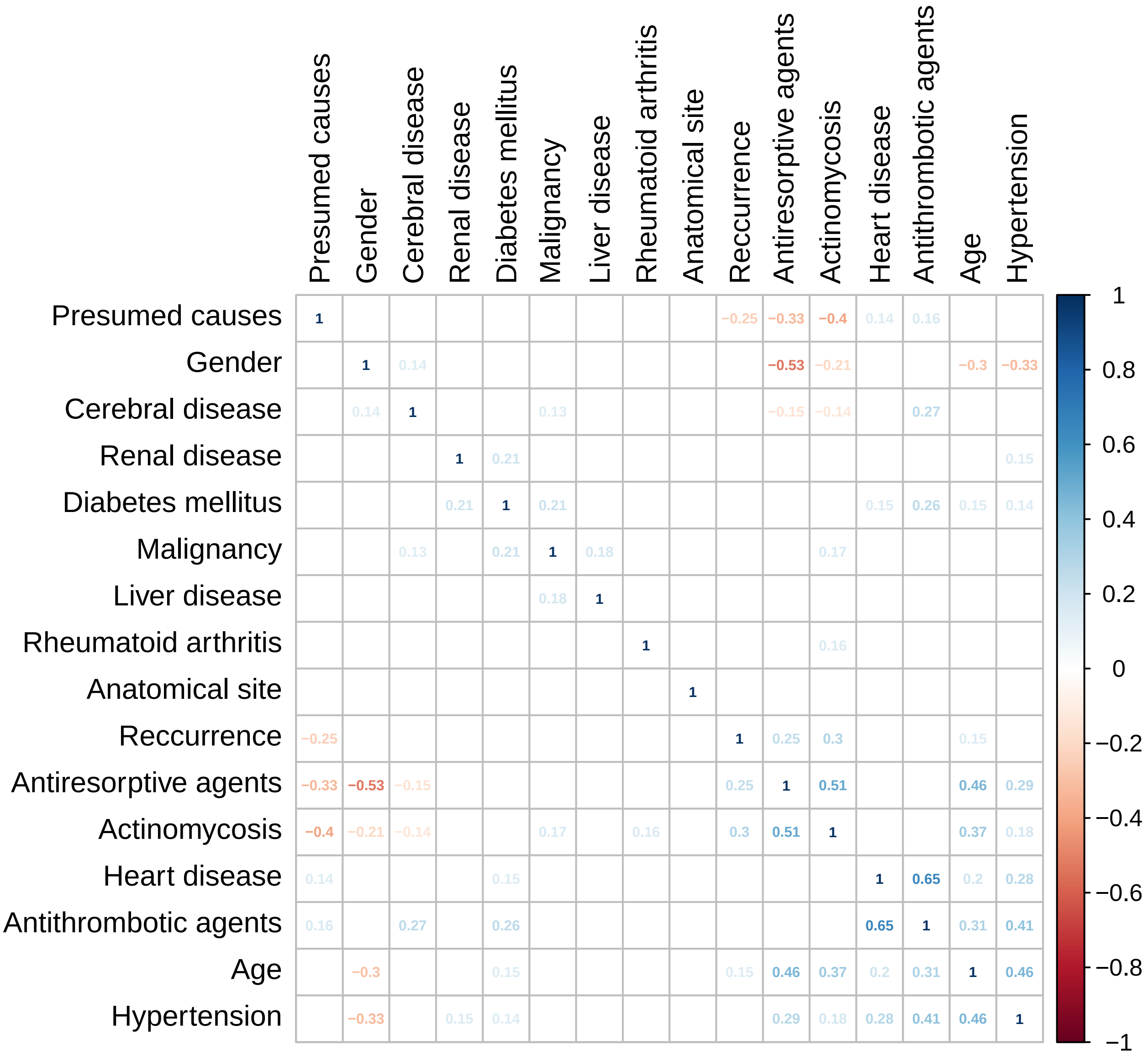

Supplementary Appendix Figure 3. Receiver operating characteristic curves plotted from testing dataset using single predictors.

AUC: area under the ROC curve, CI: confidence interval, ARA: antiresorptive agent, AS: anatomical site, MA: malignancy, HTN: hypertension, RA: reumatoid arthritis, ATA: antithrombotic agent, RD: renal disease, DM: Diabetes mellitus, LD: Liver disease, CD: Cerebral disease, PC: presumed cause

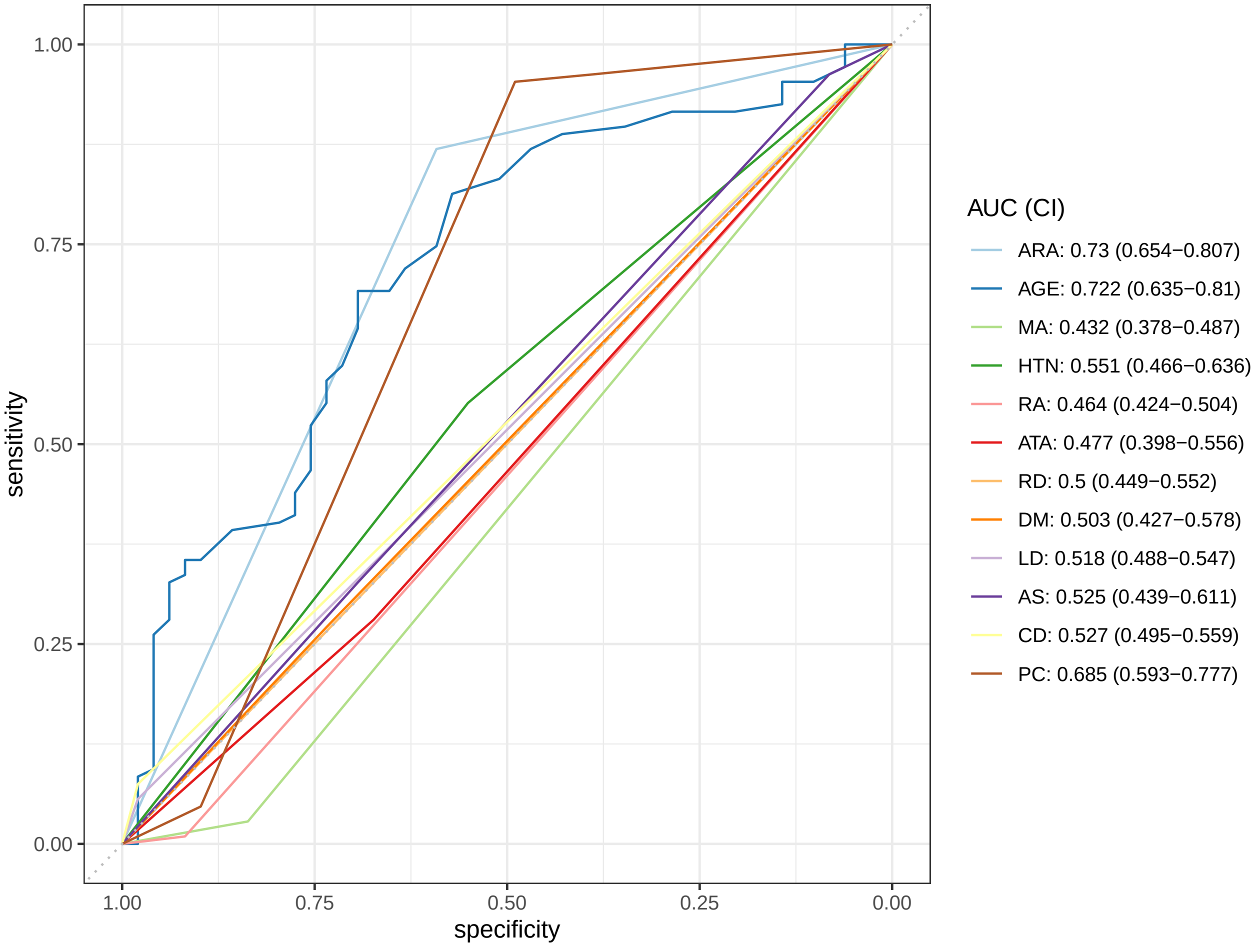

Supplementary Appendix Figure 4. Seperate ROC curves of ML models

ANN: Artificial neural network; AUC: Area under the ROC curve; CI: Confidence interval; LR: Logistic regression; ML: Machine learning; RF: Random forest, ROC: receiver operating characteristic; SVM: Support vector machine; XGB: Extreme gradient boosting.

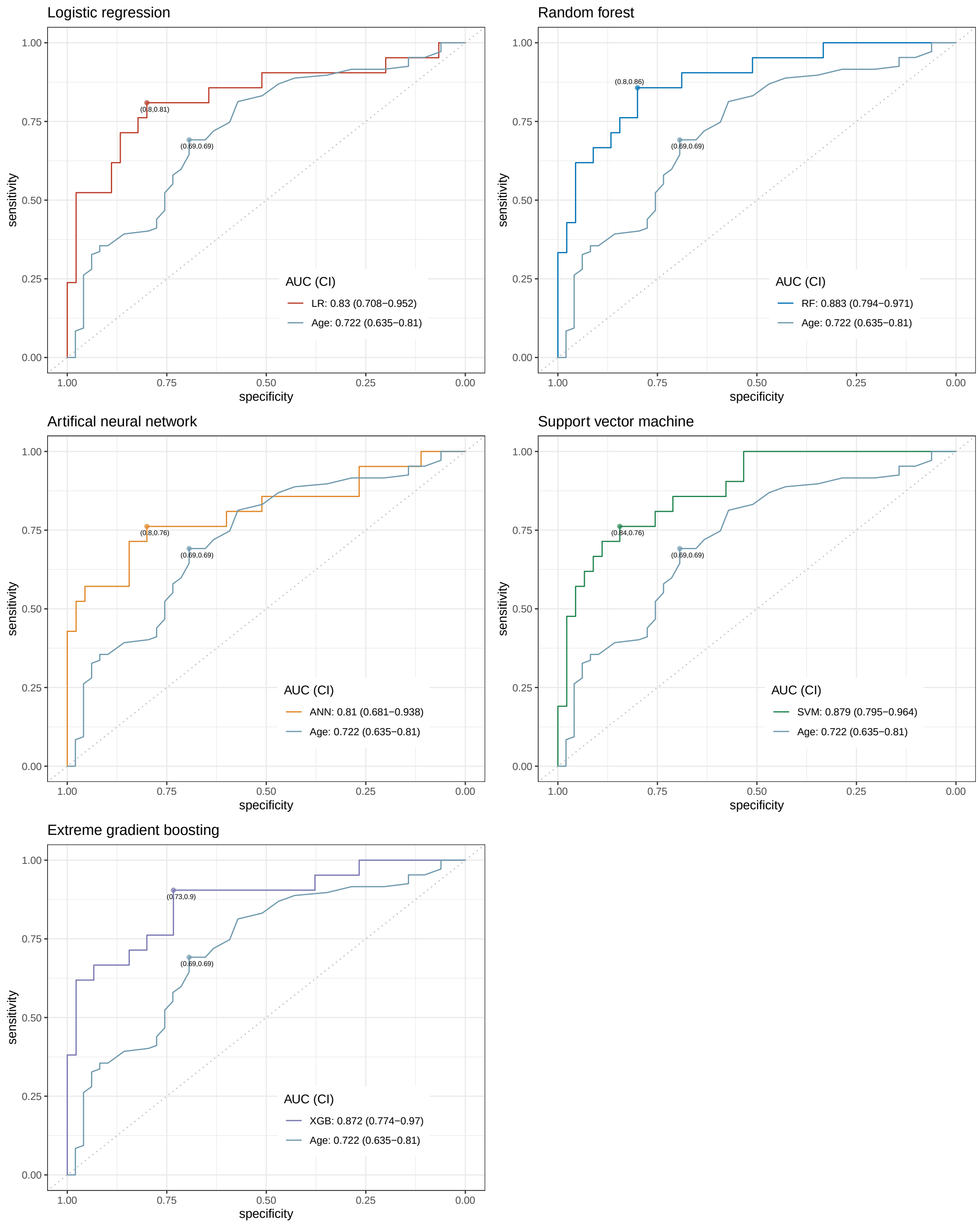

Supplementary Appendix Figure 5. Calibration plot of the prediction model.

The gray diagonal line represents the line of perfect calibration. The calibration curve of RF,SVM and XGB model is close to the diagonal reference line, indicating that the predicted and empirical probabilities are similar and that the built prediction model fits the data well.

ANN: Artificial neural network; AUC: Area under the ROC curve; LR: Logistic regression; RF: Random forest, SVM: Support vector machine; XGB: Extreme gradient boosting.

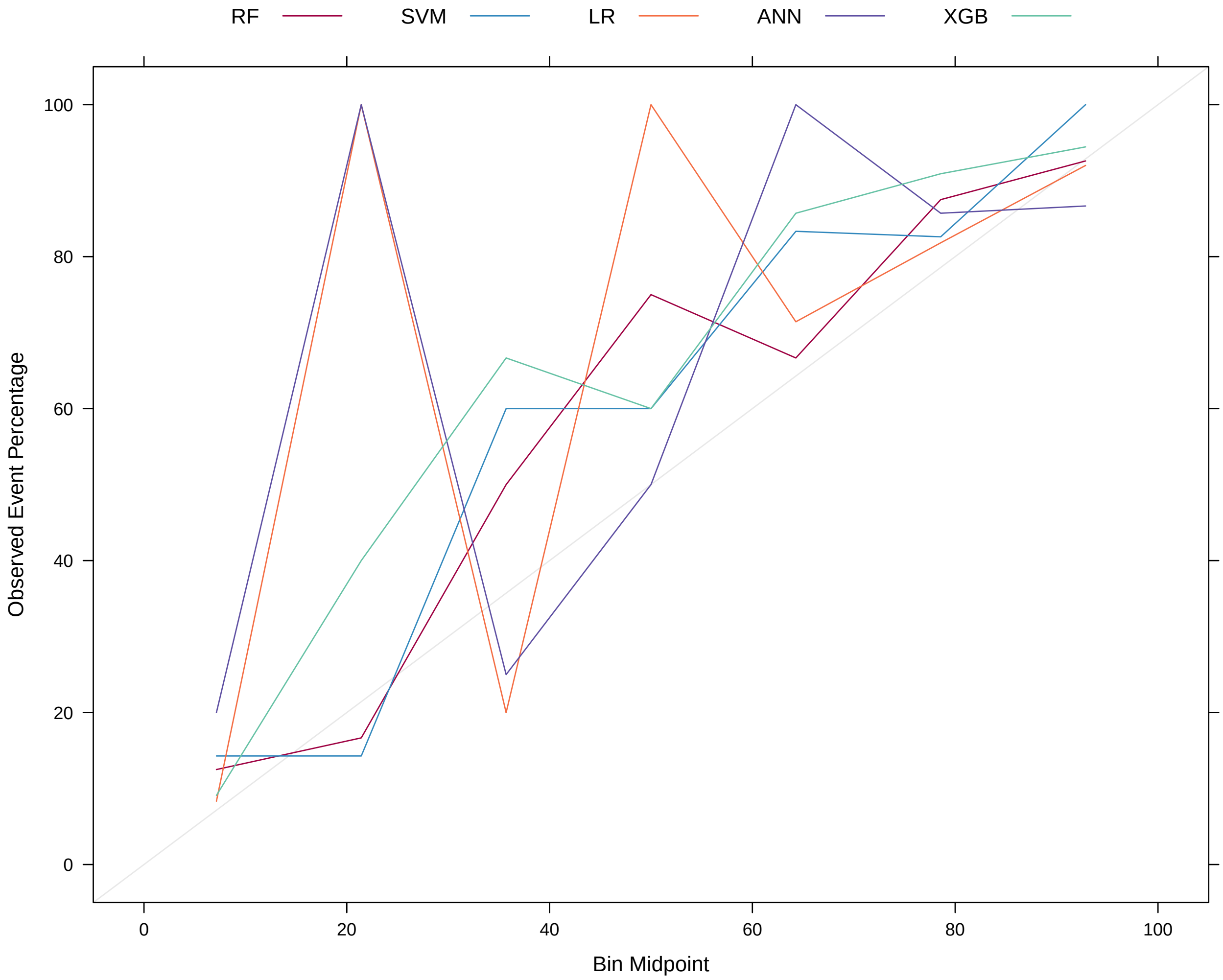

Supplementary Appendix Table 1. Optimal parameter of selected model.

Model	Method in Caret package	Optimal parameter
LR	"glm"	No tunning parameter
RF	"rf"	mtry =2, ntree = 500
SVM	"svmRadial"	Sigma = 0.05953, C = 1, kernel = 'rbf'
ANN	"nnet"	Hidden unit = 3, decay = 1
XGB	"xgbTree"	eta = 0.3,max_depth = 1, gamma = 0, min_child_weight=1, colsample_bytree = 0.6,subsample = 0.5, nround=50

Supplementary Appendix Table 2. Calculated probability of the difference between the area under the receiver operating characteristic curve.

DeLong's test was used. *$p < 0.05$

Model	RF	ANN	SVM	XGB	Age
LR	0.931	0.292	0.915	0.859	0.082
RF		0.062	0.426	0.324	0.006*
ANN			0.956	0.911	0.137
SVM				0.399	0.006*
XGB					0.013*
